# Discovery of a Positive Allosteric Modulator of Cholecystokinin Action at CCK1R in Normal and Elevated Cholesterol

**DOI:** 10.3389/fendo.2021.789957

**Published:** 2021-12-07

**Authors:** Kaleeckal G. Harikumar, Thomas Coudrat, Aditya J. Desai, Maoqing Dong, Daniela G. Dengler, Sebastian G. B. Furness, Arthur Christopoulos, Denise Wootten, Eduard A. Sergienko, Patrick M. Sexton, Laurence J. Miller

**Affiliations:** ^1^ Department of Molecular Pharmacology and Experimental Therapeutics, Mayo Clinic, Scottsdale, AZ, United States; ^2^ Drug Discovery Biology Theme, Monash Institute for Pharmaceutical Sciences, Monash University, Parkville, VIC, Australia; ^3^ ARC (Australian Research Council) Centre for Cryo-electron Microscopy of Membrane Proteins, Monash Institute for Pharmaceutical Sciences, Monash University, Parkville, VIC, Australia; ^4^ Sanford Burnham Prebys Medical Discovery Institute, La Jolla, CA, United States

**Keywords:** cholecystokinin, cholecystokinin receptor, allosteric modulation, cholesterol, obesity

## Abstract

Drugs useful in prevention/treatment of obesity could improve health. Cholecystokinin (CCK) is a key regulator of appetite, working through the type 1 CCK receptor (CCK1R); however, full agonists have not stimulated more weight loss than dieting. We proposed an alternate strategy to target this receptor, while reducing likelihood of side effects and/or toxicity. Positive allosteric modulators (PAMs) with minimal intrinsic agonist activity would enhance CCK action, while maintaining spatial and temporal characteristics of physiologic signaling. This could correct abnormal stimulus–activity coupling observed in a high-cholesterol environment observed in obesity. We utilized high-throughput screening to identify a molecule with this pharmacological profile and studied its basis of action. Compound 1 was a weak partial agonist, with PAM activity to enhance CCK action at CCK1R, but not CCK2R, maintained in both normal and high cholesterol. Compound 1 (10 µM) did not exhibit agonist activity or stimulate internalization of CCK1R. It enhanced CCK activity by slowing the off-rate of bound hormone, increasing its binding affinity. Computational docking of Compound 1 to CCK1R yielded plausible poses. A radioiodinatable photolabile analogue retained Compound 1 pharmacology and covalently labeled CCK1R Thr^211^, consistent with one proposed pose. Our study identifies a novel, selective, CCK1R PAM that binds to the receptor to enhance action of CCK-8 and CCK-58 in both normal and disease-mimicking high-cholesterol environments. This facilitates the development of compounds that target the physiologic spatial and temporal engagement of CCK1R by CCK that underpins its critical role in metabolic regulation.

## Introduction

Plasma membrane-expressed G protein-coupled receptors that are present in all excitable cells of the body are the dominant target for currently approved drugs. The type 1 cholecystokinin (CCK) receptor (CCK1R) has been identified as a potential target for drugs to treat obesity (1), one of the most prevalent and costly pathologic processes contributing to mortality and morbidity directly, as well as through co-morbidities such as diabetes mellitus and cardiovascular disease (2). The relevant CCK1R for this action is expressed outside the central nervous system on vagal afferent neurons, where activation induces satiety, thereby reducing meal size and yielding weight reduction (1, 3). Recognition that CCK, acting through CCK1R, is one of the most prominent and earliest recognized physiologic regulators of appetite (1) stimulated extensive pharmaceutical efforts to develop full agonists targeting this receptor as potential treatments for obesity. Indeed, multiple CCK1R agonists were advanced into clinical trials (3). Unfortunately, these agents did not exceed the impact of dieting on acute weight loss (4), an endpoint that has been required by the Food and Drug Administration for approval, leading to closure of these programs. Development of more potent and/or longer acting CCK1R agonists has not been encouraged, due to theoretical concerns about side effects, such as diarrhea and abdominal cramping, and potential toxicities, such as pancreatitis and trophic effects (5).

We have proposed a strategy to take advantage of the physiologic importance of CCK1R in appetite regulation that occurs in response to spatially and temporally resolved release of CCK peptides (6), circumventing the side effects and potential toxicity of full agonists, through the development of positive allosteric modulators (PAMs) of CCK action that possess little or no intrinsic agonist activity (7, 8). Such agents would not have been recognized in previously utilized screening strategies designed to identify agonists. To circumvent this limitation, we have developed a screening strategy for the identification of small-molecule PAMs. Such molecules that enhance the action of CCK under conditions where it does not directly activate the CCK1R are required to determine whether selectively targeting the physiologic pattern of receptor activation can overcome the efficacy or potential safety limitations of CCK1R agonist therapeutics.

Here, we focus on a candidate small molecule (Compound 1) that was identified in our compound screen for CCK1R PAMs. We assessed the ability of Compound 1 to enhance the action of CCK, CCK-58, the dominant form of this hormone in the circulation (9), or a partial agonist of CCK1R. We also determined the ability of Compound 1 to enhance CCK peptide action at a CCK1R variant that has been reported to mimic the behavior of the receptor in a high cholesterol environment (10), at wild type CCK1R in an enriched cholesterol environment (10), and across CCK1Rs from various species. Compound 1 is a partial agonist at very high concentrations, yet exhibits positive allosteric modulation of CCK at concentrations that do not stimulate agonist activity or internalization of the CCK1R, thereby priming the receptor for modulation at the time of physiologic release of endogenous hormone. The PAM action of Compound 1 was, at least partially, mediated by slowing the off-rate of CCK bound to CCK1R. Computational docking of Compound 1 to our recently reported high-resolution, active structure of CCK1R (11) yielded multiple potential poses. To probe the potential site of receptor interaction, we developed a photolabile probe based on the structure of Compound 1 to establish spatial approximations to the receptor. The analogue probe cross-linked to the extracellular segment of transmembrane helix 5 of CCK1R that was consistent with one of the proposed ligand poses predicted by molecular modeling, while establishing the solvent-exposed extracellular region of the receptor as the interaction domain for compound binding. Our work paves the way for the development of compounds that selectively target the physiologic spatial and temporal engagement of CCK1R by CCK peptides that underpin its critical role in metabolic regulation.

## Materials and Methods

### Materials

Synthetic CCK-26-33 (CCK-8), *D*-Tyr-Gly-[(N1e^28,31^)CCK-26-32]-phenethyl ester (CCK-OPE) (12), CCK-like radioligand *D*-Tyr-Gly-[(Nle^28,31^)CCK-26-33], and the fluorescent analogue of CCK, alexa^488^-*D*-Tyr-Gly-[(Nle^28,31^)CCK-26-33] (alexa^488^-CCK), were custom synthesized in our laboratory (13). Synthetic CCK-58 was a gift from Dr. Joseph Reeves at the University of California Los Angeles (14). NAT13-337496 from the NATx synthetic compound library (3-[(1S,2R,4S,5R)-5-ethenyl-1-azabicyclo[2.2.2]octan-2-yl]methyl-1-[3-(trifluoro methyl)phenyl]urea; here referred to as Compound 1) and its *p*-(4-hydroxybenzoyl)phenylalanine (OH-Bpa)-containing analogue (OH-Bpa-Compound 1) were synthesized by AnalytiCon Discovery GmbH (Potsdam, Germany). NAT13-333429 from the NATx synthetic compound library (methyl 3-[(([(2R,4S,5R)-5-[3-(2-methoxyphenyl)-1-methyl-1H-pyrazol-5-yl]-1-azabicyclo[2.2.2]octan-2-yl]methyl)carbamothioyl)amino]benzoate; here referred to as Compound 2) was also purchased from AnalytiCon Discovery GmbH. These compounds were purified by HPLC to homogeneity and characterized by mass spectrometry and NMR.

### Receptor Sources

The CHO-K1 (American Type Culture Collection, ATCC) cell lines expressing the rat type 1 CCK receptor (15) and the M195L rat CCK1R mutant (16), the human CCK1R (CHO-CCK1R) and Y140A human CCK1R mutant (10), and CCK1R from mouse (17) and Cynomolgus monkey (18) that were established previously were used as sources of receptor. For definitive identification of the site of labeling of this receptor, rat CCK1R mutant constructs were also prepared (i) to eliminate methionine residues in the candidate CNBr fragments that include Met^72^ to Leu (M72L), Met^121^ to Leu (M121L), and Met^225^ to Leu (M225L), and (ii) to introduce a cysteine residue to facilitate radiochemical sequencing of the receptor fragment containing the site of covalent labeling that include Ile^220^ to Cys (I220C) and Leu^223^ to Cys (L223C). These constructs were prepared using the QuikChange site-directed mutagenesis kit, following the manufacturer’s instructions (Agilent Technologies, Santa Clara, CA). The sequences of all constructs were verified by direct DNA sequencing.

A CHO-K1 cell line stably expressing the M121L mutant CCK1R was established by transfecting non-receptor-bearing CHO-K1 cells with this construct in pcDNA3.1 using Lipofectamine, as we have previously described (15). The new M72L, I220C, L223C, and M225L CCK1R mutants, as well as the previously prepared M205L mutant (16), were transfected into COS-1 cells (ATCC) using a modification of the DEAE-dextran protocol that included 10% dimethyl sulfoxide shock and treatment with 0.1 mM chloroquine diphosphate. Cells were cultured in Ham’s-F12 medium (for CHO-K1 cell lines) and in Dulbecco’s modified Eagle’s medium (for COS-1 cells) supplemented with 5% Fetal Clone II, 100 unit penicillin, and 100 µg of streptomycin.

In select experiments, we enhanced the cholesterol composition of CCK1R-expressing cells by treatment with methyl-β-cyclodextrin (MβCD)–cholesterol inclusion complex, prepared according to Pontier et al. (19), and following the procedure we previously described (10).

For photoaffinity labeling experiments, cells were harvested mechanically after being grown to confluence, and membranes enriched in plasma membranes were prepared from these cells using discontinuous sucrose gradient centrifugation, as we have previously described (15).

### Intracellular Calcium Assay

Ligand-stimulated biological activity was determined by measuring intracellular calcium responses in intact cells, as we have described (20). To examine positive allosteric effects, fixed concentrations of compounds were added simultaneously with increasing concentrations of peptide agonists. All assays were performed in duplicate and repeated at least three times in independent experiments. Concentration–response curves for peak calcium responses were analyzed and plotted as percentages of the maximal stimulation by 0.1 mM ATP using non-linear regression analysis in the Prism software suite v8.02.

### Fluorescence Polarization Assay of CCK-Like Peptide Binding Association and Dissociation

This assay was performed using alexa^488^-CCK as indicator and CHO-CCK1R membranes as source of receptor. Fluorescence polarization was measured in a Pherastar FSX instrument (BMG Labtech, Cary, NC), following the fluorescence anisotropy protocol (Ex 480 nm, Em 520), with measurements read for 0.5 s/cycle. Non-specific signals were measured under experimental conditions, in the presence of a competing saturating concentration of CCK-8 present throughout the protocol. Fluorescent ligand binding to membranes was performed by adding alexa^488^-CCK (3.2 nM) in the absence or presence of Compound 1 (10 µM) to a final volume of 200 µl of binding buffer (in KRH buffer, pH 7.4 with 0.2% bovine serum albumin) in a 96-well black Opti-plate (Perkin Elmer) for a minimum of 75 cycles. After reaching a plateau, dissociation of the fluorescent CCK ligand from the receptor was initiated by adding a saturating concentration of unlabeled CCK-8 (1 µM) in the absence or presence of Compound 1 (10 µM) (at 50 cycles) and collecting the FP signal for another 25 cycles.

### Peptide Radioiodination

The CCK-like ligand (*D*-Tyr-Gly-[(Nle^28,31^)CCK-26-33]) and OH-Bpa-Compound 1 were radioiodinated using the procedure we have described (21). The radioiodinated products were purified by reversed-phase HPLC to yield specific radioactivities of ~2000 Ci/mmol (21).

### Receptor Binding

The radioligand competition-binding assay was used to determine binding affinity and receptor density of CCK receptor constructs expressed on intact cells, as we have described (10). All assays were performed in duplicate and repeated at least three times in independent experiments. The impact of allosteric modulators on an orthosteric ligand was calculated using the operational model of allosterism (22).

### Receptor Internalization

The impact of the small-molecule ligand on internalization of the CCK1R was studied in CHO-CCK1R cells. The assay quantified receptors on the cell surface after various treatments by incubating the cells at 4°C with a fluorescent CCK analogue, alexa^488^-CCK, fixing it in place with 2% paraformaldehyde, and imaging. Cell surface fluorescence was visualized using Zeiss Axiovert 200M inverted epifluorescence microscope with YFP filter setting (Ex 480/dichroic mirror Q515lp/Em 525, 40 × 1.4 numerical aperture). Images were captured using ORCA-12ER camera (Hamamatsu, Bridgewater, NJ) with QED *In Vivo* (version 2.039) acquisition software (Media Cybernetics, Silver Spring, MD).

The treatments were performed at 37°C in phosphate-buffered saline (PBS), pH 7.4, containing 0.1 mM MgCl_2_ and 0.08 mM CaCl_2_, with the experimental agent, Compound 1 (10 µM), and the negative control representing the buffer, and the positive control representing CCK-8 (1 µM). A time course of the treatments was studied. After the treatment period, cells were washed with ice-cold PBS and then incubated further with alexa^488^-CCK (50 nM) for 60 min at 4°C to occupy all the available cell surface receptors.

### Photoaffinity Labeling

Membranes (~50 µg) prepared from cells were incubated with ∼0.1 nM radioiodinated OH-Bpa-Compound 1 in the absence or presence of 10 µM nonradioactive Compound 1 for 1 h at room temperature in the dark. This was exposed to UV irradiation with 3,500-Å lamps for 30 min at 4°C in a Rayonet photochemical reactor (Southern New England Ultraviolet, Hamden, CT) (23). Membranes were washed twice with ice-cold photoaffinity labeling medium and solubilized in 200 µl of this medium containing 1% Nonidet P-40 and 0.1% SDS overnight at 4°C. Supernatants were incubated with 20 µl of wheat germ agglutinin gel bead slurry (EY Laboratories, San Mateo, CA) overnight at 4°C, washed, and loaded on 10% SDS-polyacrylamide gels. Radiolabeled bands were visualized by autoradiography. Apparent molecular masses were determined by interpolation on a plot of mobility of ProSieve protein standards (Cambrex, Rockland, ME) versus the log values of their apparent masses.

### Peptide Mapping

Larger-scale photolabeling was performed using 100 µg of plasma membrane aliquots, following the same procedure described above. After electrophoresis, radioactive bands of interest were excised from gels, as described previously (24). For selected experiments, receptor samples were deglycosylated by treatment with peptide-N-glycosidase F from Prozyme (Hayward, CA). Purified radiolabeled CCK1R samples were cleaved with 5 mg of CNBr in 200 µl of 70% formic acid containing 0.1% SDS, and the products of cleavage were analyzed on 10% Bis-Tris NuPAGE gels (Invitrogen, Carlsbad, CA) using MES running buffer under reducing conditions. The radiolabeled bands were visualized by autoradiography, and their apparent molecular weights were determined by interpolation on a plot of the mobilities of the appropriate SeeBlue Plus2 multicolored standards (Invitrogen) *versus* the log values of their apparent masses.

### Radiochemical Sequencing

To identify the specific residue covalently labeled with OH-Bpa-Compound 1, radiochemical sequencing of the fragment identified above, containing the site of labeling, was performed. In brief, the CCK1R fragment Gln^206^-Met^225^ from CNBr cleavage of the L220C or I223C CCK1R mutant was purified to radiochemical homogeneity and was coupled to *N*-(2-aminoethyl-1)-3-aminopropyl glass beads through sulfhydryl groups within Cys^220^ or Cys^223^ within the mutant receptor constructs. Cycles of Edman degradation were repeated manually in a manner that has been previously reported in detail (25), and the radioactivity released in each cycle was quantified in a γ-spectrometer.

### Molecular Modeling

The recently solved CCK-occupied CCK1R complexed with Gs (11) was used as a starting point for the modeling since it had the best ligand-binding region resolution of the structures reported in that study. All molecules except for the receptor CCK1R (chain R) were deleted and CCK1R was prepared using the Maestro (Schrodinger, LLC) protein preparation wizard as follows: adding hydrogens, adding missing side chains, determining protonation states and side-chain orientation based on hydrogen bond optimization, removing the waters, and minimizing the structure using the OPLS4 force field. Compound 1 was sketched in Maestro and its 3D conformation was prepared with Ligprep (Schrodinger, LLC). A CCK1R-apo docking grid was prepared using the CCK-8 ligand to identify the binding pocket region. Compound 1 was docked using Glide (26) into the CCK1R apo grid and the top scoring pose was used to form a CCK1R–Compound 1 complex. This complex was used as the starting point to the induced fit docking (IFD) protocol from Schrodinger (27). Docking of Compound 1 was carried out using the extended IFD protocol on the Glide-generated CCK1R–Compound 1 complex. Interaction fingerprints were generated in Maestro on the resulting 41 receptor–ligand complexes using all interaction types. These complexes were then clustered based on interaction fingerprints using the average linkage method.

### Statistical Analysis

Differences between groups of experiments were evaluated using the Mann–Whitney test, with *p* < 0.05 considered to be significant.

## Results

### Characteristics of the Small-Molecule PAM

**Figure 1:**
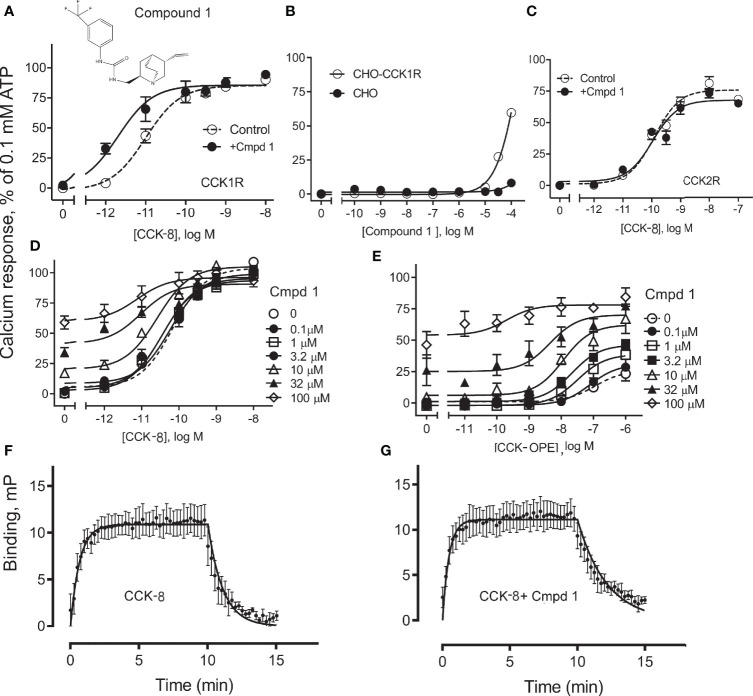
Effect of Compound 1 on CCK-stimulated intracellular calcium responses at human CCK1R and CCK2R. Shown in **(A)** is the chemical structure of Compound 1 and the impact of 10 µM of this compound on intracellular calcium responses to CCK-8 in CHO-CCK1R cells that express high levels of this receptor. Compound 1 shifted the CCK-8 concentration–response curve to the left, acting as a PAM (**Table 1**). **(B)** shows that Compound 1 did not directly stimulate an intracellular calcium response in the CHO-CCK1R cells when used in concentrations as high as 10 µM, and stimulated submaximal responses when used in 32 µM and 100 µM concentrations, while even these very high concentrations of Compound 1 did not elicit significant intracellular calcium responses at the non-receptor-bearing parental CHO cells. **(C)** shows that under similar conditions, Compound 1 had no modulatory effect on the ability of CCK-8 to stimulate intracellular calcium responses in CHO-CCK2R cells expressing the highly related type 2 CCK receptor (**Table 1**). **(D, E)** illustrate the ability of various concentrations of Compound 1 to affect concentration–response curves for CCK-8 and CCK-OPE to stimulate intracellular calcium responses in CHO-CCK1R cells. These assays were also performed with very high concentrations of Compound 1 to allow calculation of allosteric constants. Under these conditions, PAM effects of this compound are obvious, with most marked shifts observed for the partial agonist, CCK-OPE. Values are expressed as percentages of the intracellular calcium responses to maximal stimulation achieved by 0.1 mM ATP and data points represent the means ± S.E.M. of data from a minimum of three independent experiments performed in duplicate. **(F, G)** show curves reflecting fluorescent CCK binding association and dissociation in the absence **(F)** and presence **(G)** of 10 μM Compound 1. The off-rate of bound peptide is significantly slowed in the presence of Compound 1. Values reflect means ± S.E.M. of data from a minimum of three independent experiments.

Compound 1 was identified in a high-throughput screening project looking for small-molecule compounds that possess positive allosteric modulatory activity to enhance CCK action at the CCK1R, while possessing minimal endogenous agonist activity, a profile we have previously proposed for possible use in obesity to safely reduce appetite and lead to sustainable weight loss (7). The structure and pharmacological characterization of this candidate molecule are shown in **Figure 1**. In panel A, we illustrate the ability of this compound to enhance CCK-8 stimulated intracellular calcium responses in CHO-CCK1R cells, shifting the hormone concentration–response curve to the left (pEC_50_: Control, 11.0 ± 0.1; in the presence of 10 µM Compound 1, 11.7 ± 0.2; *p* < 0.01, *n* = 5). Panel B shows that Compound 1 did not directly stimulate an intracellular calcium response in the CHO-CCK1R cells when used in concentrations as high as 10 µM, and stimulated submaximal responses when used in 32 µM and 100 µM concentrations, while even these very high concentrations of Compound 1 did not elicit significant intracellular calcium responses at the non-receptor-bearing parental CHO cells. Panel C shows that Compound 1 did not modulate CCK-8-stimulated intracellular calcium responses in CCK2R-expressing cells under similar conditions despite an equivalent level of receptor expression, and affinity for ^125^I-CCK-8, for the CCK1R and CCK2R cell lines (human CCK1R/human CCK2R: pKi for CCK-8, 8.9 ± 0.1/9.2 ± 0.1; receptor number × 10^5^ sites/cell 0.6 ± 0.1/0.6 ± 0.1). Thus, Compound 1 is a selective PAM of CCK1R-mediated mobilization of intracellular calcium. Panels D and E illustrate the impact of increasing concentrations of Compound 1 to 100 µM on the CCK1R concentration–response curves of CCK-8 and the partial agonist, CCK-OPE, respectively. Of note, at concentrations above 10 µM, Compound 1 was also a weak agonist. These data were quantified with an operational allosteric model (22) to derive estimates of Compound 1 (ligand B) affinity for the free receptor (pK_B_), intrinsic efficacy (LogTau_B_), and composite cooperativity factor (Logαβ) with CCK-8 at CCK1R; pK_B_ = 3.6, LogTau_B_ = 0.74, and Logαβ = 1.6, *n* = 4 (~40-fold enhancement of CCK-8 signaling). The modulatory shifts induced by Compound 1 were more pronounced for the partial agonist, CCK-OPE; however, the effect could not be accurately quantified using the operational model, since data did not approach saturation of the modulatory effect within the concentration range assessed.

We also probed the mechanism of the PAM activity of Compound 1 through assessment of the effect of the compound on the binding off-rate of fluorescently labeled CCK (alexa^488^-CCK). The kinetics of CCK association and dissociation are illustrated in panels F and G. While the rates of association of the CCK-like ligand in the absence and presence of 10 µM Compound 1 (expressed as K_on_ × 10^8^ M^−1^ min^−1^) were not statistically different from each other, 1.4 ± 1.0 and 4.2 ± 1.2, respectively (*p* = 0.3, *n* = 4), Compound 1 significantly slowed the off-rate of the peptide (expressed as K_off_ min^−1^), 1.3 ± 0.3 and 0.4 ± 0.1, respectively, for absence and presence of Compound 1 (*p* = 0.03, *n* = 4). Thus, a reduction in the kinetics of unbinding of CCK peptide is a contributor to the PAM activity of Compound 1.

**Figure 2:**
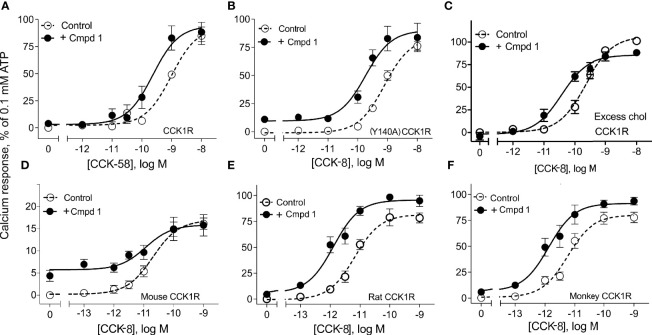
Effects of Compound 1 on CCK-stimulated intracellular calcium responses at CCK1R in normal and high cholesterol and at various species of CCK1R. Shown are the effects of 10 µM of Compound 1 on CCK-peptide-stimulated intracellular calcium responses (**Table 1**). Human CCK1R constructs are studied in **(A–C)**, with mouse CCK1R in **(D)**, rat CCK1R in **(E)**, and Cynomolgus monkey CCK1R in **(F)**. Data in **(A)** show impact on stimulation by CCK-58, the dominant form of this hormone in the human circulation. Data in **(B)** show impact on a CCK1R mutant, (Y140A)CCK1R, previously shown to mimic this receptor in a high cholesterol environment. This cell line exhibited higher affinity for CCK than WT CCK1R [human CCK1R/human (Y140A)CCK1R: pKi for CCK-8, 8.9 ± 0.1/10.1 ± 0.3], with similar levels of receptor expression [human CCK1R/human (Y140A)CCK1R: receptor number × 10^5^ sites/cell, 0.6 ± 0.1/0.5 ± 0.1]. Data in **(C)** show impact on effects of CCK-8 at wild-type CCK1R in cells in which cholesterol composition has been augmented. All of these are CHO cell lines stably expressing CCK1R. Values are expressed as percentages of the intracellular calcium responses to maximal stimulation achieved by 0.1 mM ATP and data points represent the means ± S.E.M. of data from a minimum of three independent experiments performed in duplicate.

**Figure 2** illustrates the utility of Compound 1 in modulation of the predominant circulating form of CCK, CCK-58, and modulation of CCK peptide response across different species homologues of the CCK1R, as well as under conditions that mimic high cholesterol states that attenuate functional responses to CCK peptides. Compound 1 enhanced the potency of CCK-58 relative to the control (*p* < 0.05, *n* = 5) (panel A), as well as responses to CCK-8 in a receptor variant shown to mimic a high cholesterol environment, (Y140A)CCK1R (*p* < 0.05, *n* = 3) (panel B), or to the wild-type CCK1R expressed in a high-cholesterol environment (panel C) (*p* < 0.05, *n* = 9) (**Table 1**). Because some allosteric ligands are dependent on receptor epitopes that are not as highly conserved as those responsible for orthosteric ligand binding, we also assessed Compound 1 modulation of CCK-8 responses for CCK1R from other species, including mouse (panel D), rat (panel E), and Cynomolgus monkey (panel F). While the rat and Cynomolgus monkey CCK1R-expressing cell lines had similar receptor density to the human, the mouse receptor-bearing line exhibited lower expression (human/rat/mouse/Cynomolgus monkey CCK1R: pKi for CCK, 8.9 ± 0.1/9.1 ± 0.3/8.6 ± 0.2/8.6 ± 0.2; receptor number × 10^5^ sites/cell, 0.6 ± 0.1/0.7 ± 0.1/0.1 ± 0.1*/0.6 ± 0.1). At the rat and monkey CCK1R, Compound 1 was a robust positive allosteric modulator, enhancing the action of CCK-8 to stimulate intracellular calcium to a similar extent to that seen with the human CCK1R (**Table 1**); however, at the mouse CCK1R, no significant enhancement was observed. Of interest, at the mouse CCK1R, there was higher endogenous agonist activity of Compound 1, relative to the CCK-8 response, than observed in the other species, in spite of the relatively low level of receptor expression in this cell line.

**Table 1:** Pharmacological parameters of ligand-induced intracellular calcium responses in various CCK receptor-bearing cell lines in the absence and presence of Compound 1 and Compound 2.

Receptor–Ligand	pEC_50_ for CCK	*p*-value, relative to control
**CCK1R – CCK-8**		
Control	11.0 ± 0.1 (11)	
+ 10 µM Cmpd 1	11.7 ± 0.2 (5)**	0.001
+ 10 µM Cmpd 2	11.4 ± 0.2 (5)*	0.04
**CCK2R–CCK-8**		
Control	9.9 ± 0.1 (5)	
+ 10 µM Cmpd 1	10.0 ± 0.2 (5)	0.84
**CCK1R–CCK-58**		
Control	9.1 ± 0.1 (10)	
+ 10 µM Cmpd 1	9.9 ± 0.3 (5)*	0.02
**Mouse CCK1R–CCK-8**		
Control	10.7 ± 0.1 (10)	
+ 10 µM Cmpd 1	10.8 ± 0.2 (4)	0.5
**Rat CCK1R–CCK-8**		
Control	11.1 ± 0.2 (8)	
+ 10 µM Cmpd 1	11.9 ± 0.1 (4)*	0.01
**Monkey CCK1R–CCK-8**		
Control	11.2 ± 0.1 (10)	
+ 10 µM Cmpd 1	11.8 ± 0.2 (6)*	0.03
**CCK1R WT excess chol–CCK-8**		
Control	9.7 ± 0.1 (10)	
+ 10 µM Cmpd 1	10.5 ± 0.2 (9)**	0.001
**(Y140A)CCK1R–CCK-8**		
Control	9.1 ± 0.1 (5)	
+ 10 µM Cmpd 1	9.7 ± 0.1 (3)*	0.04
**CCK1R–CCK-8**		
Control	11.0 ± 0.1 (6)	
+ 10 µM OH-Bpa-Cmpd 1	11.8 ± 0.2 (6)**	0.008

Data represent means ± S.E.M. of duplicate determinations in “n” independent experiments (numbers displayed in parentheses). Differences between control and the presence of small-molecule compounds were evaluated using the Mann–Whitney test. *p < 0.05; **p < 0.01.

### Internalization of CCK1R

**Figure 3:**
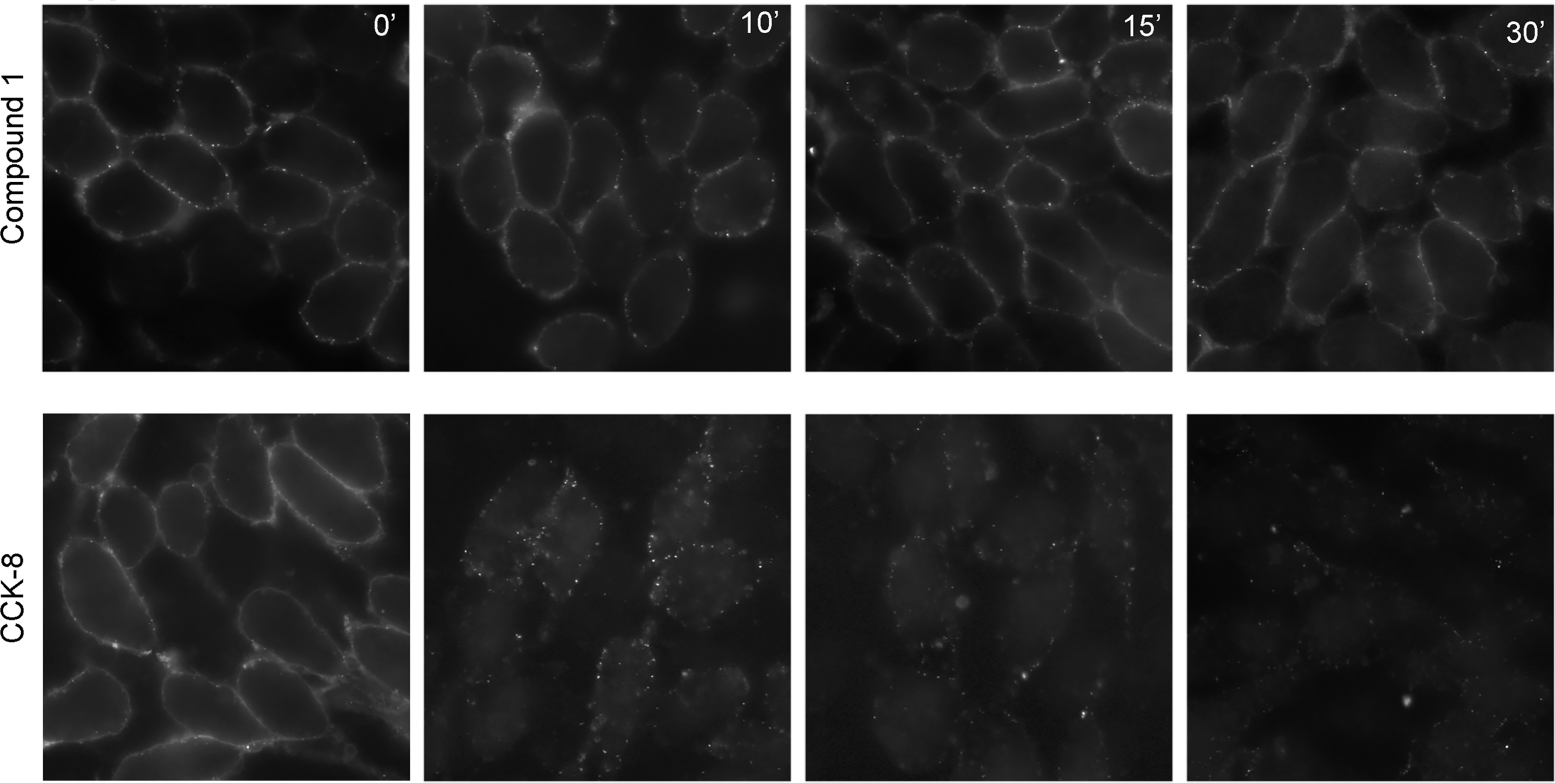
Impact of Compound 1 on CCK1R internalization. Shown are time courses of CCK1R internalization, reflected as loss of cell surface receptor, stimulated by exposure to 10 μM Compound 1 (top series) or 1 μM CCK-8 (bottom series). After exposure of the CHO-CCK1R cells to the ligands at 37°C, cells were cooled to 4°C and surface receptor was quantified by probing with alexa^488^-CCK. Compound 1 had no significant impact on the cadre of cell surface receptor, while CCK-8 stimulated prompt receptor internalization. Shown are the representative images from three independent experiments.

For PAMs with little or no intrinsic efficacy to be effective, it is important that they do not stimulate internalization, in the absence of endogenous agonist peptides. Shown in **Figure 3**, 1 µM CCK-8 stimulates rapid internalization of CCK1R, whereas 10 µM Compound 1 did not alter the surface cadre of CCK1R following exposure for up to an hour. Thus, at concentrations of Compound 1 that enhance CCK peptide signaling, but engender little intrinsic agonism, the compound does not alter the available cell surface receptor pool for response to endogenous stimulation.

### Molecular Modeling of Compound 1-Bound CCK1R

**Figure 4:**
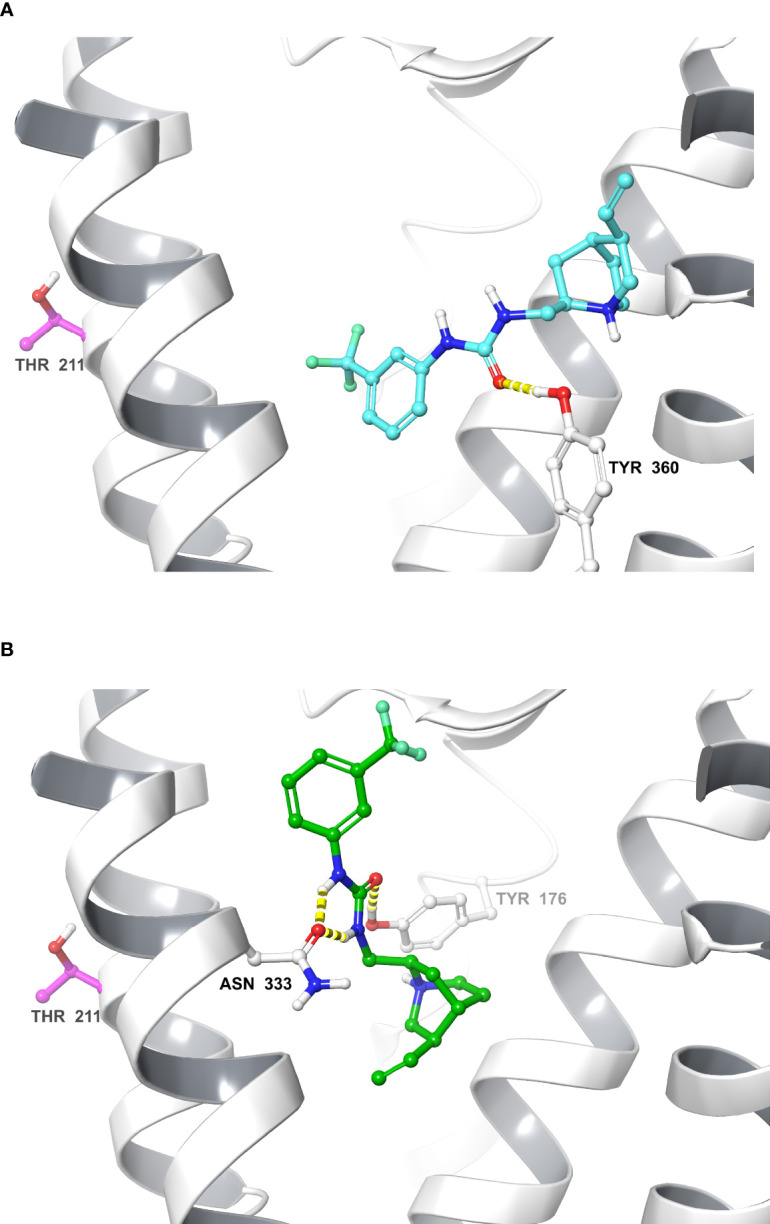
Molecular models of Compound 1 bound to the CCK1R. Shown are molecular models of the docking of Compound 1 to the active CCK1R (11), following removal of the peptide agonist. The two poses depicted represent the two principal converged clusters based on their interaction patterns: the largest cluster **(A)**, and the cluster containing the highest scoring IFD pose **(B)**. TM7 and ECL3 ribbons are not displayed for clarity. Interactions are denoted in yellow (hydrogen bond). The residue Thr^211^ is represented in purple.

The IFD protocol generated 41 CCK1R–Compound 1 complexes that were clustered based on their ligand–receptor interaction. These complexes converged into two main clusters that represent two alternate orientations for Compound 1; the largest cluster contained 15 IFD complexes and the second largest cluster contained 12 IFD complexes, and also included the overall highest scoring IFD complex. We selected for further analysis the top IFD scoring complexes from the largest cluster (**Figure 4A**) and the second largest cluster (**Figure 4B**), the highest IFD scoring complex.

### Photoaffinity Labeling of CCK1R With the OH-Bpa-Compound 1

**Figure 5:**
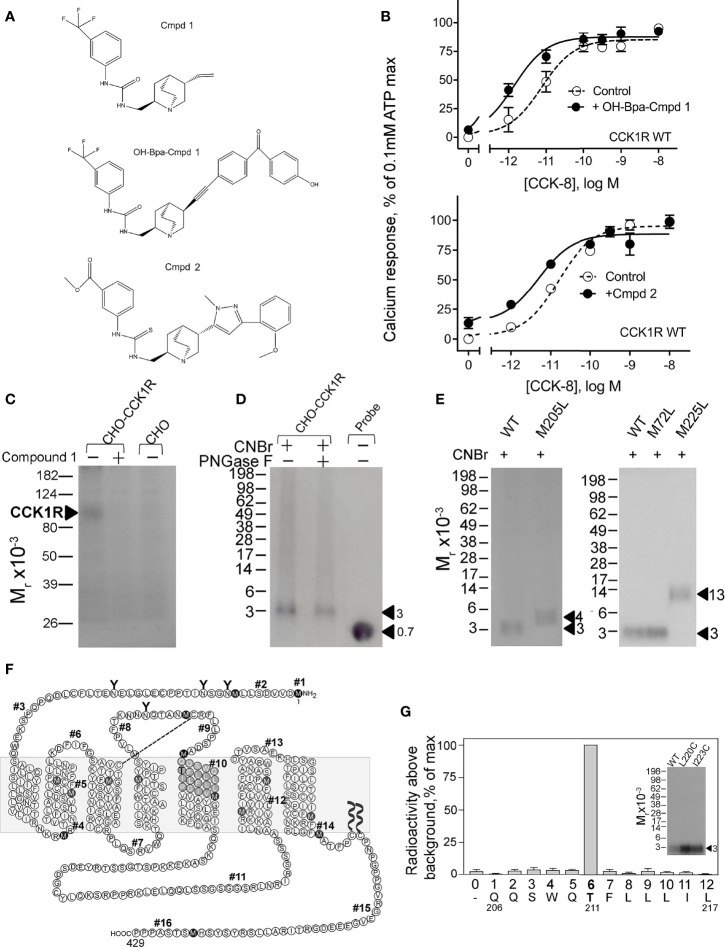
Characterization of OH-Bpa-Compound 1 and identification of its site of labeling CCK1R. Shown in **(A)** are the chemical structures of compounds. **(B)** shows the impact of 10 µM OH-Bpa-Compound 1 and Compound 2 on intracellular calcium responses to CCK-8 in CHO-CCK1R cells. Values are expressed as percentages of the intracellular calcium responses to maximal stimulation by 0.1 mM ATP and data points represent means ± S.E.M. of data from a minimum of three independent experiments performed in duplicate. **(C)** shows a representative autoradiograph of membranes from cells labeled with the radioligand and separated on a 10% SDS-PAGE gel. This resulted in labeling a band migrating at *M_r_* = 85–95,000 representing CCK1R, with labeling inhibited by unlabeled Compound 1. No labeling was observed for non-receptor-bearing CHO cell membranes. **(F)** shows a diagram that illustrates the theoretical fragments resulting from CNBr cleavage of wild type rat CCK1R, with predicted molecular mass of each fragment shown in **Table 2**. **(D)** shows a representative autoradiograph of 10% Bis-Tris NuPAGE gels used to separate products of CNBr cleavage of the CCK1R band labeled with OH-Bpa-Compound 1. This non-glycosylated band migrated at approximate *M_r_* = 3,000. **(E)** illustrates representative autoradiographs of 10% Bis-Tris NuPAGE gels used to separate products of CNBr cleavage of labeled CCK1R constructs in which specific sites of CNBr cleavage were modified. The region of covalent labeling was definitively identified as fragment #10, shown by appropriate changes in migration when Met residues at either end of this fragment were replaced by Leu. Cleavage of the labeled receptor M205L mutant yielded a band migrating at approximate *M*_r_ = 4,200 and that of the labeled receptor M225L mutant yielded a band migrating at approximate *M_r_* = 13,000. **(G)** shows that adding Cys residues to fragment #10 to allow its covalent attachment to beads for Edman degradation sequencing did not interfere with the affinity labeling (inset) and the profile of elution of radioactivity from Edman degradation sequencing of the purified Gln^206^-Leu^217^ fragment resulting from CNBr cleavage of these constructs. The peak in eluted radioactivity was consistently observed in cycle 6 in four independent experiments. This corresponds to covalent attachment of the OH-Bpa-Compound 1 probe to residue Thr^211^ at the top of TM5 of the CCK1R.

To refine our understanding of the molecular basis of the interaction of Compound 1 with CCK1R, we prepared a photolabile radioiodinatable analogue that contains a OH-Bpa (**Figure 5A**). The design of this probe was based on structure–activity considerations identified in the primary high-throughput screen in which a compound related to Compound 1 also exhibited PAM activity, Compound 2. This molecule included an extension of similar size to the photolabile benzoyl-phenylalanine group to be used in photoaffinity labeling. OH-Bpa-Compound 1 demonstrated equivalent cooperativity to Compound 1 for enhancing the action of CCK-8 at CCK1R (**Figure 5B**). Similarly Compound 2 enhanced the action of CCK-8 to stimulate a calcium response in CCK1R cells (**Figure 5B**).

We radioiodinated OH-Bpa-Compound 1 and purified it to radiochemical homogeneity for use in photoaffinity labeling studies. We utilized rat CCK1R constructs for this part of our work, since we have previously used this species CCK1R extensively in our earlier photoaffinity labeling studies, and we already have a battery of rat CCK1R mutants as tools for site identification of labeling (28). As noted above, we also confirmed that Compound 1 has similar cooperativity with CCK peptides at rat and human CCK1R (**Figure 2**). Radioiodinated OH-Bpa-Compound 1, specifically and saturably, covalently labeled the CCK1R (**Figure 5C**). This labeling was competitively inhibited by 10 µM Compound 1, and the control, non-receptor-bearing CHO-K1 cell membranes did not get labeled (**Figure 5C**).

We have extensively used CNBr to resolve peptide fragments of GPCRs that are labeled by photolabile cross-linking, due to its quantitative efficiency of cleaving at the carboxyl side of Met residues within a protein sequence (25). CCK1R contains 15 Met residues, and theoretically, CNBr cleavage of this receptor yields 16 fragments ranging in molecular mass from 0.1 to 9.9 kDa, with two fragments containing potential sites of *N*-linked glycosylation (**Figure 5F** and **Table 2**). Here, CNBr cleavage of the CCK1R resolved the site labeled by the OH-Bpa-Compound 1 probe to a band migrating at approximate *M_r_* = 3,000 that did not shift further after deglycosylation with PNGase F (**Figure 5D**). This band migrated in a position distinct from the free radioiodinated probe (~*M_r_* = 700). Considering the non-glycosylated nature and size of the free iodinated probe (671 Da), the mass of the candidate fragment would be expected to be approximately 2.3 kDa. There are four CNBr cleavage products that could account for this mass: fragment #4 (Arg^73^-Met^89^, ~1.9 kDa) at the bottom of TM2, #6 (Pro^96^-Met^121^, ~3.0 kDa) spanning ECL1, #8 (Thr^174^-Met^195^, ~2.5 kDa) at the amino-terminal half of ECL2, and #10 (Gln^206^-Met^225^, ~2.4 kDa) at the top of TM5 (**Table 2**). To definitively identify which of these fragments contained the site of labeling, five CCK1R mutants in which a naturally occurring Met residue in positions that border the candidate fragments was replaced by a Leu residue were chosen for further analysis, specifically, M72L, M121L, M195L, M205L, and M225L. Each of these mutant CCK1R constructs was specifically labeled by the OH-Bpa-Compound 1 probe followed by CNBr cleavage with the results shown in **Table 2** and **Figure 5E**. Mutation of Met^205^ or Met^225^, but not the other methionines, increased the size of the fragment, demonstrating that CNBr fragment #10, at the top of TM5 (Gln^206^-Met^225^), contained the site of labeling. To identify the specific receptor residue in the Gln^206^-Met^225^ fragment labeled by the probe, two cysteine mutants were prepared, Ile^220^ (I220C) or Leu^223^ (L223C), for use in radiochemical sequencing. Both mutants were also labeled by the radioiodinated OH-Bpa-Compound 1 probe with CNBr cleavage fragments of *M_r_* = 3,000, similar to the CNBr fragment from the wild type CCK1R (**Figure 5G**). Radiochemical sequencing of the purified CNBr fragments from the I220C or L223C mutant receptor revealed a radioactive peak in cycle 6, corresponding to the labeling of receptor residue Thr^211^ at the top of TM5 (**Figure 5G**). This site of labeling is compatible with the Compound 1 pose from IFD docking illustrated in **Figure 4B**.

**Table 2:** Identification of the receptor CNBr cleavage fragment containing the site of labeling using CCK1R mutants.

CNBr fragment # (diagram in **Figure 5F**)	CNBr fragment sequence	M.W. (Da)	Calculated M.W. of fragment with probe	Candidates after cleavage of WT receptor	Eliminated fragment #6 after cleavage of M121L	Eliminated fragment #8 after cleavage of M195L	Eliminated fragment #4 after cleavage of M72L	Confirmed fragment #10 containing site of labeling after cleavage of M205L	Further confirmed fragment #10 containing site of labeling after cleavage of M225L
1	M^1^	149	820						
2	D^2^-M^9^	891	1,562						
3	N^10^-M^72^	7,087+	7,758+						
4	R^73^-M^89^	1,893	2,564	Y	Y	Y	Expected: 9,651+ Observed: ~3,000 (see **Figure 5D**)		
5	L^90^-M^95^	729	1,400						
6	P^96^-M^121^	2,982	3,653	Y	Expected: 9,396 Observed: ~3,000				
7	G^122^-M^173^	5,743	6,414						
8	T^174^-M^195^	2,528+	3,199+	Y	Y	Expected: 4,351+ Observed: ~3,000			
9	C^196^-M^205^	1,152	1,823						
10	Q^206^-M^225^	2,361	3,032	Y	Y	Y	Y	Expected: 4,182 Observed: ~4,200 (see **Figure 5E**)	Expected: 12,940 Observed: ~13,000 (see **Figure 5E**)
11	V^226^-M^315^	9,908	10,579						
12	L^316^-M^328^	1,596	2,267						
13	P^229^-M^374^	5,129	5,800						
14	N^375^-M^383^	1,168	1,839						
15	A^384^-M^422^	4,251	4,922						
16	S^423^-P^429^	656	1,327						

## Discussion

The power, safety, and selectivity of positive allosteric modulators of hormonal action, together with their potential to augment physiological signaling that is not possible with full agonists acting at the same receptors (29), provide novel opportunities for disease intervention. Such molecules have now been approved by the Food and Drug Administration (30). CCK acts at the vagal afferent CCK1R to mediate postprandial satiety; however, while full agonist drugs of this receptor have stimulated weight reduction in patients, they have not achieved the primary end point of exceeding the effectiveness of acute dieting in clinical trials for obesity (31). Moreover, application of more potent and longer-duration agonists is limited by side effects (8), as well as by the theoretical possibility of toxicities such as induction of pancreatitis or even pancreatic cancer due to the trophic activity of such molecules (5, 32, 33). In contrast, a CCK1R PAM with minimal intrinsic agonist activity could provide a safer and more efficacious approach for targeting this receptor by enhancing the temporally finite, and spatially resolved, physiological short duration of action of endogenous CCK released after a meal (7, 8). Importantly, PAMs have the potential to correct the aberrant stimulus–activity coupling of CCK action at the CCK1R observed in a high-cholesterol microenvironment, such as exists in some obese patients (34, 35).

Traditional efforts to develop drugs acting at the CCK1R have focused on the discovery of compounds with high intrinsic efficacy for receptor activation. High-throughput screens to identify such compounds, by definition, would have missed molecules that possessed minimal or no intrinsic agonist activity, such as PAMs that could augment endogenous signaling. In contrast, Compound 1 that is characterized in the current study was identified in a high-throughput screen for compounds that augment CCK-8 peptide signaling, but had little or no intrinsic agonist activity at the 10 µM concentration used in the screen. In the current study, we demonstrate that Compound 1 exhibits key pharmacological properties that are pre-requisite to its potential suitability as a scaffold for PAM drug development, including low intrinsic activity, selectivity, maintenance of activity across CCK1R from species relevant to preclinical obesity drugs, augmentation of the predominant circulating form of CCK, and activity in disease-mimicking conditions. Quantitation of the cooperative effect of Compound 1 on CCK peptide signaling using an operational allosteric model indicated that the compound augmented signaling by ~40-fold, while this effect was selective for CCK1R over the closely related CCK2R. These data also revealed that Compound 1 has only low affinity (~100 μM) for CCK1R in the absence of peptide, which was consistent with its low potency as a partial agonist. Analysis of the effect of Compound 1 on peptide binding kinetics demonstrated that it slowed the off-rate of CCK, consistent with affinity cooperativity that is thermodynamically reciprocal between the two ligands (36), being a key component of its mechanism of action. Importantly, the PAM activity of Compound 1 was equivalent for CCK-58 that is the predominant circulating form of CCK, and for augmentation of CCK signaling at rat and monkey CCK1R that represent critical animal models for preclinical assessment of potential anti-obesity drug (8). PAM activity at the mouse CCK1R was less robust, with also the potential for higher intrinsic efficacy, suggesting that there may be some species selectivity in the mode of action. However, further studies are required as the cell line expressing mouse receptors had lower expression than for the other species.

An additional potential advantage of PAMs with limited intrinsic efficacy is the ability to circulate without driving internalization of the receptor that could blunt physiological signaling. In the current work, Compound 1 did not cause receptor internalization at 10 μM, a concentration that robustly augments peptide signaling but has minimal intrinsic signaling efficacy. Since Compound 1 has only low affinity for the receptor, it will have low occupancy unless the peptide is bound (~1% at 10 μM), providing a mechanism for such compounds to circulate but not modulate receptor function or cell surface accessibility in the absence of physiological stimulus.

Recent work has demonstrated that CCK stimulus–activity coupling can be affected by the cholesterol composition of the plasma membrane (37) and that there is a broad spectrum of sensitivity to CCK across the population (37). We have long understood that gallbladder muscularis CCK1R exhibited aberrant CCK stimulus–activity coupling in patients with cholesterol gallstones (38). In cells with excess cholesterol, CCK bound with increased affinity, yet elicited a reduced calcium response (39). This could be corrected by extracting cholesterol from such cells (40). Recently, an assay was developed in which wild-type CCK1R could be delivered *ex vivo* to buffy coat cells using a viral vector, permitting quantitation of the sensitivity of such cells to CCK (37). There was a spectrum of cholesterol levels expressed in such cells that correlated with obesity, and CCK sensitivity was reduced in the cells with the highest cholesterol composition (37). This demonstrated that the effect of membrane composition extended well beyond the biliary tree with cells bathed in cholesterol-rich bile. In another recent important study, CCK-stimulated gallbladder contraction was quantified *in vivo* using HIDA scans (41). In studying those patients with normal complete ejection fractions and no evidence of biliary obstruction, there was still a spectrum of CCK responsiveness observed (41). Like the *ex vivo* studies (37), here the reduced CCK sensitivity also was correlated with excess body mass. These observations provide another possible explanation for the failure of CCK1R agonists in previous clinical trials for obesity treatment. It is likely that at least some of the subjects included in those studies exhibited this aberrant CCK stimulus–activity coupling. Importantly, in the current study, Compound 1 was equally effective in augmenting CCK peptide signaling at both the wild-type CCK1R in a high-cholesterol environment (42) and a mutant CCK1R previously shown to mimic this receptor in a high-cholesterol environment (10). Thus, Compound 1-like drugs could also correct the aberrant stimulus–response coupling observed in diseased patients.

To gain insight into the site of action of Compound 1, we performed computational docking studies that were further supported by the development and application of a labeled photoactive analogue of the compound. Since Compound 1 is a partial agonist when bound to CCK1R, we used an active, G protein-coupled structure of CCK1R for docking (10) where the peptide agonist had been removed. Analysis of the top scoring poses revealed clustering in one of two (flipped) orientations that were energetically feasible, which predicted that Compound 1 would bind to the extracellular face of the receptor, partially overlapping with a region that would be occupied by the amino terminus of the peptide agonist in the solved structure (11) (**Figure 4**). Analysis of photo-crosslinking of the analogue revealed selective labeling of Thr^211^ at the top of TM5 of CCK1R that supported the highest-scoring IFD complex (**Figure 4B**) where the ethene moiety of Compound 1, which was effectively replaced by the photolabile Bpa in the analogue, was oriented towards Thr^211^. These data, together with the pharmacological data illustrating slowing of peptide off-rate kinetics, are consistent with an extracellular site of action for both the allosteric modulation and intrinsic agonism.

Collectively, our work demonstrates that molecules with the key characteristics required for CCK1R PAM therapeutic development exist and thus opens the path for evolution of compounds for proof-of-concept evaluation of targeting augmentation of physiological CCK1R signaling for the treatment of obesity.

## Data Availability Statement

The datasets presented in this study can be found in the **Supplementary Material**.

## Author Contributions

KH: acquisition of data, analysis and interpretation of data, statistical analysis, and drafting manuscript. TC: acquisition of data, analysis and interpretation of data, statistical analysis, and drafting manuscript. AD: acquisition of data, analysis and interpretation of data, and statistical analysis. MD: acquisition of data, analysis and interpretation of data, and statistical analysis. DD: acquisition of data, analysis and interpretation of data, and statistical analysis. SF: study supervision, analysis, and interpretation of data. AC: study concept and design and interpretation of data. DW: study concept and design, analysis and interpretation of data, study supervision, and drafting of manuscript. ES: study concept and design, study supervision, analysis, and interpretation of data. PS: study concept and design, study supervision, analysis and interpretation of data, and drafting of manuscript. LM: study concept and design, study supervision, analysis and interpretation of data, drafting of manuscript, and obtained funding. All authors contributed to the article and approved the submitted version.

## Funding

This work was supported by a grant from the National Institutes of Health, R01 DK115402.

## Conflict of Interest

The authors declare that the research was conducted in the absence of any commercial or financial relationships that could be construed as a potential conflict of interest.

## Publisher’s Note

All claims expressed in this article are solely those of the authors and do not necessarily represent those of their affiliated organizations, or those of the publisher, the editors and the reviewers. Any product that may be evaluated in this article, or claim that may be made by its manufacturer, is not guaranteed or endorsed by the publisher.

## Abbreviations

CCK, cholecystokinin; CCK1R, type 1 CCK receptor; CCK2R, type 2 CCK receptor; IFD, induced fit docking; MβCD, methyl-β-cyclodextrin; PAM, positive allosteric modulator.
